# Two-Session Radiosurgery for Large Primary Tumors Affecting the Brain

**DOI:** 10.7759/cureus.7850

**Published:** 2020-04-27

**Authors:** Eduardo E Lovo, Kaory C Barahona, Fidel Campos, Victor Caceros, Carlos Tobar, William A Reyes

**Affiliations:** 1 Radiosurgery, International Cancer Center, Diagnostic Hospital, San Salvador, SLV; 2 Radiation Oncology, International Cancer Center, Diagnostic Hospital, San Salvador, SLV; 3 Radiation Oncology, International Cancer Center, San Salvador, SLV

**Keywords:** brain tumors cns tumors, photon stereotactic radiosurgery, brain stereotatic radiosurgery

## Abstract

Introduction

Surgery is an option for patients with large, symptomatic primary tumors affecting the brain. However, surgery might not be suitable for all tumors, especially those located in sensitive areas such as the pineal region and the hypothalamus. Single-session stereotactic radiosurgery (SRS) might not provide an adequate dose for long-term local control due to the initial tumor volume and the involvement of radiation sensitive organs at risk (OARs). Two-session radiosurgery has been described as a feasible strategy for dose escalation in large secondary brain tumors. This report describes a series of patients treated upfront with two-session radiosurgery for primary tumors affecting the brain.

Materials and methods

From May 2017 to January 2020, eight patients with primary tumors affecting the brain were treated with two-session radiosurgery due to either an initial large tumor volume or tumor localization and the involvement of OARs. The response was assessed by imaging and clinical evaluations.

Results

A total of eight patients were treated, nine tumors were treated with two-session radiosurgery, four patients had tumors in the pineal region (50%), and the rest were in the hypothalamic region (25%) or elsewhere. The mean tumor volume for the first SRS session was 15 mL (range 5.2 to 51.6 mL), the mean prescription dose was 13 Gy, and the timespan between both sessions was 30 days (range, 30 to 42 days). During the second session, tumor volume was reduced to 73.6% (range, -20% to 98.7%) of the original dimension, mean tumor volume was 5 mL (range, 0.1 to 17.8 ml), mean prescription dose for the second session was 16.2 Gy estimated by time, dose, and fractionation and by bioequivalent dose under alpha-beta values often to be equivalent to a single dose of 15.8 Gy. Doses to the OARs for the optic pathway were equivalent to a single maximum dose of 9.75 Gy (range, 7.12 to 10.92), and to the brainstem, the equivalent was a maximum dose of 12.3 Gy (range, 5.6 to 15.07).

At last follow-up, at a mean of 336.5 days (range, 65 to 962 days), seven patients were alive, five tumors had a partial response (PR), and three had stable disease in accordance to Response Evaluation Criteria in Solid Tumors (RECIST) criteria. One patient died 435 days after treatment, the Karnofsky Performance Status (KPS) was 90 at the first session, 90 at the second session, and was maintained at last follow-up. No adverse radiation effects were reported.

Conclusions

Two-stage SRS proved to be a safe method to escalate dose in proportionately large volume primary brain tumors whose histology is expected to have a quick biological response to radiation. Longer follow-up is needed to determine the long-term effectiveness by tumor subtypes of two-stage SRS in the same manner as it has been proven in single session SRS series in smaller tumor volumes.

## Introduction

Two- and three-session stereotactic radiosurgery (SRS), also called staged radiosurgery, has been described in the treatment of voluminous metastatic lesions affecting the brain as a form of dose escalation [1-8]. The rationale behind two- and three-stage SRS for metastases is to space fractions by 30-day or 15-day intervals, respectively, to total 30 Gy to a planning target volume (PTV). The lesion becomes smaller in the time between fractions, thus allowing higher doses with steeper dose gradients on the second or third session. The regular practice of SRS usually limits the initial dose deliverable to a tumor by its size; lower doses to large brain tumors are well known to affect local control as opposed to smaller tumors given higher doses [9-11].

Current alternatives to managing large primary tumors affecting the brain in eloquent areas such as those surrounding the pineal region or the hypothalamus include surgery, biopsy, fractionated radiation therapy, or chemotherapy. In addition to these issues, these tumors are relatively rare and very heterogeneous in their histology and behavior. Due to their central location in the brain and their size, they are usually abutting and displacing eloquent structures such as the thalamus and tectum as well as encasing important vascular structures, making surgical resection, or even stereotactic biopsy, challenging. Open surgery can be associated with a risk of neurological deficit, and residuals usually warrant further treatment with radiation [12-16]. Radiosurgery has been an alternative to treat small tumor volumes with usual marginal dose prescriptions around 15 Gy for the treatment of pineal or other primary tumors in the brain; many studies report different local tumor control and survival [17-22]. In large tumors of the hypothalamus or in the pineal region that deform sensitive structures such as the visual pathway, thalamus, and brainstem, covering the lesion with the usual recommended dose can be difficult since the prescription dose is higher than the recommended tolerance levels of the OARs.

The rationale of two-session radiosurgery in large primary tumors or those located in sensitive areas is the same as applied to large brain metastases. The sum of both doses provides for dose escalation that allows the treatment to achieve the dose recommended for a smaller volume than could have been managed in a single fraction scheme.

We describe the technical feasibility and our initial series of patients using a two-session radiosurgery strategy that allowed for a significant reduction of the original tumor volume to deliver a second radiosurgical treatment. This second treatment, in summation by bioequivalent dose (BED) and time-dose-fraction (TDF) calculations, could deliver 15 Gy or more as equivalent dose (EQD2) in a single fraction.

## Materials and methods

The present study is a retrospective series of eight patients with primary tumors affecting the brain that were treated with a two-session radiosurgery technique. Treatment was performed between May 2017 and January 2020 using a rotating gamma-ray unit known as Infini™ (Masep Medical Company, Shenzhen, China). This study was approved by the ethics committee of our institution; all patients provided informed consent for treatment. All patients were evaluated by a team of neurosurgeons and radiation oncologists to determine if further surgery of any form was feasible or necessary before radiosurgical treatment. For those patients amenable to surgery, the case was discussed with family members and primary caregivers who opted for radiosurgery over surgery considering the potential risks of an open technique. All patients were informed that if their neurological symptoms worsened or did not improve after radiosurgery, immediate surgery would be recommended. Images were taken during the next treatment, at 30 days, and every three months thereafter to evaluate response or progression.

**Figure 1:**
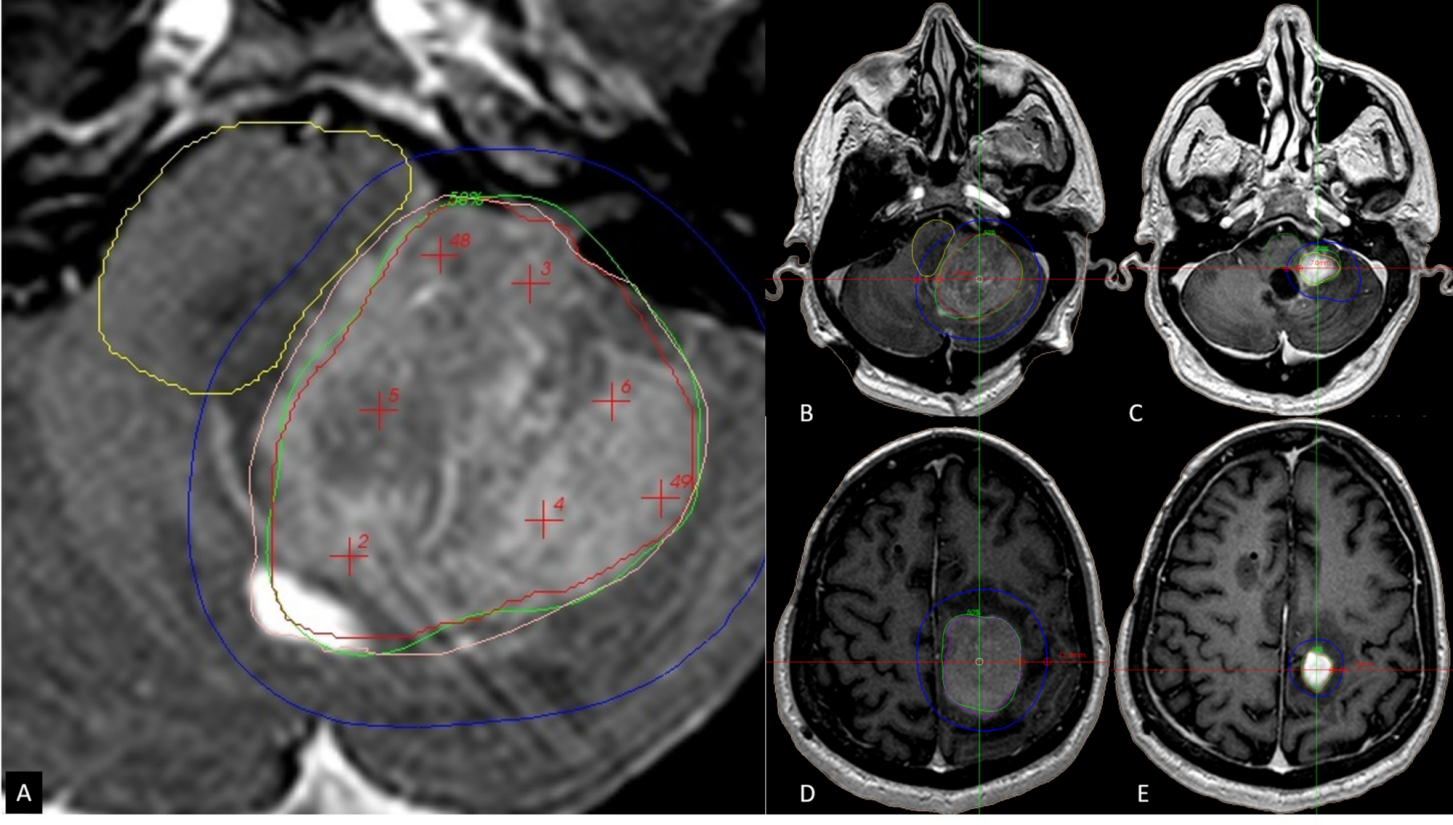
Patient number seven presenting with two large lesions from primary central nervous system lymphoma A. The tumor (GTV) is outlined in pink and the brainstem in yellow. Inside the GTV, there is a PTV outlined in red and purposely separating the PTV from the area of tumor contact to the brainstem. The green isodose line corresponds to 50% and the prescription dose for the first session of 11 Gy. The brainstem received 10 Gy max dose and a mean dose of 4.4 Gy. B. Axial T1 gadolinium-enhanced image showing in green the 50% isodose line that corresponds to 11 Gy and the blue isodose line that corresponds to the dose gradient (25% of the prescription dose 5.5 Gy). The medial measurement between the isodose curves of healthy tissue is 11.5 mm. C. Axial T1 gadolinium-enhanced image at 30 days during the patient’s second session. Now the green isodose corresponds to 11.5 Gy to the 50% line and again the blue line to the dose gradient that is now 7 mm. The dose in the brainstem was, on the second session, a max dose of 9.9 Gy with a mean dose of 3.3Gy, after TDF calculations and to a BED of a single-fraction SRS brainstem received max dose of 13.3 Gy mean dose of 10.6Gy. D. A second lesion at the left parietal lobe is shown. The green isodose line corresponds to 50% of the prescription dose of 12 Gy for the first session, a blue isodose line is 6 Gy, corresponding to the dose gradient. The measurement of healthy tissue is 12.3mm. E. At 30 days, the new target volume is being prescribed 13 Gy to the 50% line, and the dose gradient corresponding to the blue isodose line is now 6.5 Gy to an area measuring 4.8 mm. BED, bioequivalent dose; GTV, gross tumor volume; PTV, planning target volume; SRS, stereotactic radiosurgery; TDF, time, dose, and fractionation.

In brief, the two-session radiosurgery technique has been described by our group elsewhere [1]. All patients underwent radiosurgery on an outpatient basis, with 8-mg doses of dexamethasone administered intravenously on the day of treatment, followed by 2-mg doses of oral dexamethasone every eight hours for one week or as needed, until the patient was clinically improved and stable. Stereotactic frame placement was done by a neurosurgeon. Patients underwent magnetic resonance imaging (MRI) with a 1.5-Tesla Avanto™ (Siemens Corporation. Erlangen, Germany), usually consisting of only one volumetric T1 single dose contrast of 1.0 to 1.5-mm slice thickness with no spacing of the region of interest. A typical T2 constructive interference in steady state of the same region with the same slice thickness was acquired to help delimitate OARs. Organs at risk, including the visual pathway and brainstem, were contoured by neurosurgery, and the PTV was usually contoured by radiation therapy specialists; neurosurgeons usually drew the PTV smaller than the gross total volume (GTV), especially in areas in proximity to the OARs (Figure 1).

The intended prescription dose in a BED with both fractions to a single-session dose was >15 Gy. All patients were prescribed to the 50% isodose line and scheduled for subsequent treatments based on a two-session radiosurgery protocol. Patients were evaluated at 48 hours and one week after initial treatment. Thirty days after the initial treatment, patients were reevaluated neurologically for symptoms and proceeded with the second session of radiosurgery. Stereotactic MRI acquisition was repeated as described earlier, and a new plan configured based on the new volume of the lesion.

## Results

Of the eight patients treated, six were men and two were women. The average age was 28 years (range, 6-64). Four had tumors in the pineal region, two had pinealocytomas, one had germinoma, and one had unknown histology. Two patients had drop metastases in the hypothalamic region: one from medulloblastoma and the other from an anaplastic ependymoma. Of the remaining two patients, one had chondrosarcoma of the right cavernous sinus, and the other was a patient with AIDS who had two large lesions from a primary central nervous system lymphoma (Table 1), a total of nine tumor were treated in two-session radiosurgery protocol.

**Table 1:** Patient and treatment characteristics KPS, Karnofsky Performance Status; IDL, isodose line; RECIST, Response Evaluation Criteria in Solid Tumors; PR, partial response; SD, stable disease; SRS, stereotactic radiosurgery.

N	Sex	Age	Diagnosis	Number of tumors	Anatomic localization	Initial KPS	Tumor volume (cm³) at the first session of SRS	First SRS prescription dose at 50% IDL	Mean dose	Timespan of the second session	Tumor volume (cm³) at the second session of SRS	Percentage of reduction	Second SRS prescription dose at 50% IDL	Mean dose	KPS at second SRS	RECIST Last follow up	Alive	Follow-up
1	M	21	Drop metastasis medulloblastoma	4	Hypothalamus	70	5.2	10	13.26	30.00	0.4	92.31%	13	18.83	80	PR	No	403
2	F	64	Unknown	1	Pineal region	80	21.8	12	15.56	32.00	17.8	18.35%	12	15.85	100	SD	Yes	962
3	F	40	Chondrosarcoma	1	Right cavernous sinus	70	15	15	19.1	30.00	12.2	18.67%	15	19.38	80	SD	Yes	886
4	M	35	Pineocytoma	1	Pineal region	80	14.4	14	18.3	30.00	5	65.28%	14	17.79	90	PR	Yes	833
5	M	12	Germinoma	1	Pineal region	90	20.2	10	13.6	30.00	0.7	96.53%	13	16.41	100	PR	Yes	270
6	M	11	Pineocytoma	1	Pineal region	70	7.5	13	18.88	42.00	0.1	98.67%	10	13.72	100	PR	Yes	252
7	M	42	Primary CNS Lymphoma	2	Left parietal	20	51.6	12	15.65	30.00	3.3	93.60%	13	16.26	50	PR	Yes	71
					Left pontine angle		43.3	11	15.34	30.00	11.4	73.67%	11.5	15.48				
8	M	6	Drop metastases Anaplastic Ependymoma	1	Hypothalamus	90	5.5	10	13.14	42.00	6.6	-20.00%	11	14.25	100	SD	Yes	65
Total/ Mean		28				90	15	12	15.56	30.00	5	73.67%	13	16.26	95			336.5

The mean tumor volume at the first session was 15 mL (range, 5.2 to 51.6 mL), the mean prescription dose was 13 Gy (range, 10 to 15 mL), mean dose was 15.5 Gy (range, 13.1 to 19.1 Gy), and PTV coverage was 90% with a conformity number of 0.83. The time between both sessions was 30 days (range, 30 to 42 days). During the second session, tumor volume had reduced to 73.6% (range, -20 to 98.7%) of the original dimension, the mean tumor volume was 5 mL (range, 0.1 to 17.8 mL), the mean prescription dose was for the second session was 16.2 Gy (range, 13.7 to 19.3 mL). The sum of both sessions was EQD2 to a single dose to be 15.8 Gy, according to TDF and BED under alpha-beta values that were normalized to 10 for all lesions except the chondrosarcoma, which was calculated with an alpha-beta of two. The EQD2 single-dose equivalent for the mean dose was 20.8 Gy (Table 2).

**Table 2:** Equivalent prescription dose calculations for PTV Calculations are based on TDF and EQD2 and BED for prescription dose and mean dose. *alpha/beta of 2. BED, bioequivalent dose; EQD2, equivalent dose; TDF, time, dose, and fractionation; PTV, planning target volume.

Equivalent prescription dose (Gy) to monofraction using TDF	TDF prescription dose	EQD2 prescription dose (Gy) alpha/beta 10	BED prescription dose (Gy) alpha/beta 10	Equivalent mean dose (Gy) to monofraction using TDF	TDF mean dose	EQD2 Mean dose (Gy) alpha/beta 10	BED mean dose (Gy) alpha/beta 10
14.85	75.57	30.75	36.9	20	119.27	50	60
16	84.88	34.67	41.6	20.89	127.64	53.77	64.73
20	119.64	110*	220*	25.9	177.71	180.65*	361.3*
18.7	107.59	44.72	53.67	24.22	160.27	69.07	82.88
14.3	71.76	28.96	34.75	18.94	109.82	45.68	64.81
15.8	83.41	33.97	40.76	22.67	144.82	61.72	74.06
16.6	89.07	36.8	44.16	21.22	130.75	55.21	66.25
15	76.3	31.25	37.5	20.58	124.71	52.44	62.93
13.7	66.43	27.06	32.47	17.85	100.27	41.43	49.71

Dose to the OARs for the tumors near the optic pathway using TDF, BED, and EQD2 maximum dose was 9.75 Gy (range, 7.12 to 10.92), mean dose was 6.3 Gy (range, 3.6 to 7), and the dose to the brainstem was maximum dose of 12.3 Gy (range, 5.6 to 15.07), mean dose was 3.91 Gy (range, 3.8 to 10.6; Table 3).

**Table 3:** Equivalent prescription dose calculations for organs at risk Calculations are based on TDF and EQD2 and BED for prescription dose and mean dose. BED, bioequivalent dose; Dmax, maximum dose; EQD2, equivalent dose; TDF, time, dose, and fractionation; PTV, planning target volume.

Equivalent Dmax, Brainstem to monofraction	TDF	EQD2 Brainstem to monofraction	TDF	Equivalent Dmax, Optic pathway to monofraction	TDF	Equivalent Dmax, Optic pathway to monofraction	TDF
9	34.87	2.6	5.31	9.75	39.52	7.02	23.89
13.11	62.39	4.3	11.19	NA	NA	NA	NA
12.22	55.98	4.55	12.23	10.95	47.25	6.3	20.23
15.07	77.29	3.87	9.56	NA	NA	NA	NA
12.47	57.71	3.95	9.81	NA	NA	NA	NA
12.02	54.58	1.65	2.56	NA	NA	NA	NA
13.3	63.94	10.65	45.18	NA	NA	NA	NA
5.67	17.16	2.75	5.65	7.12	24.39	3.6	8.57

**Figure 2:**
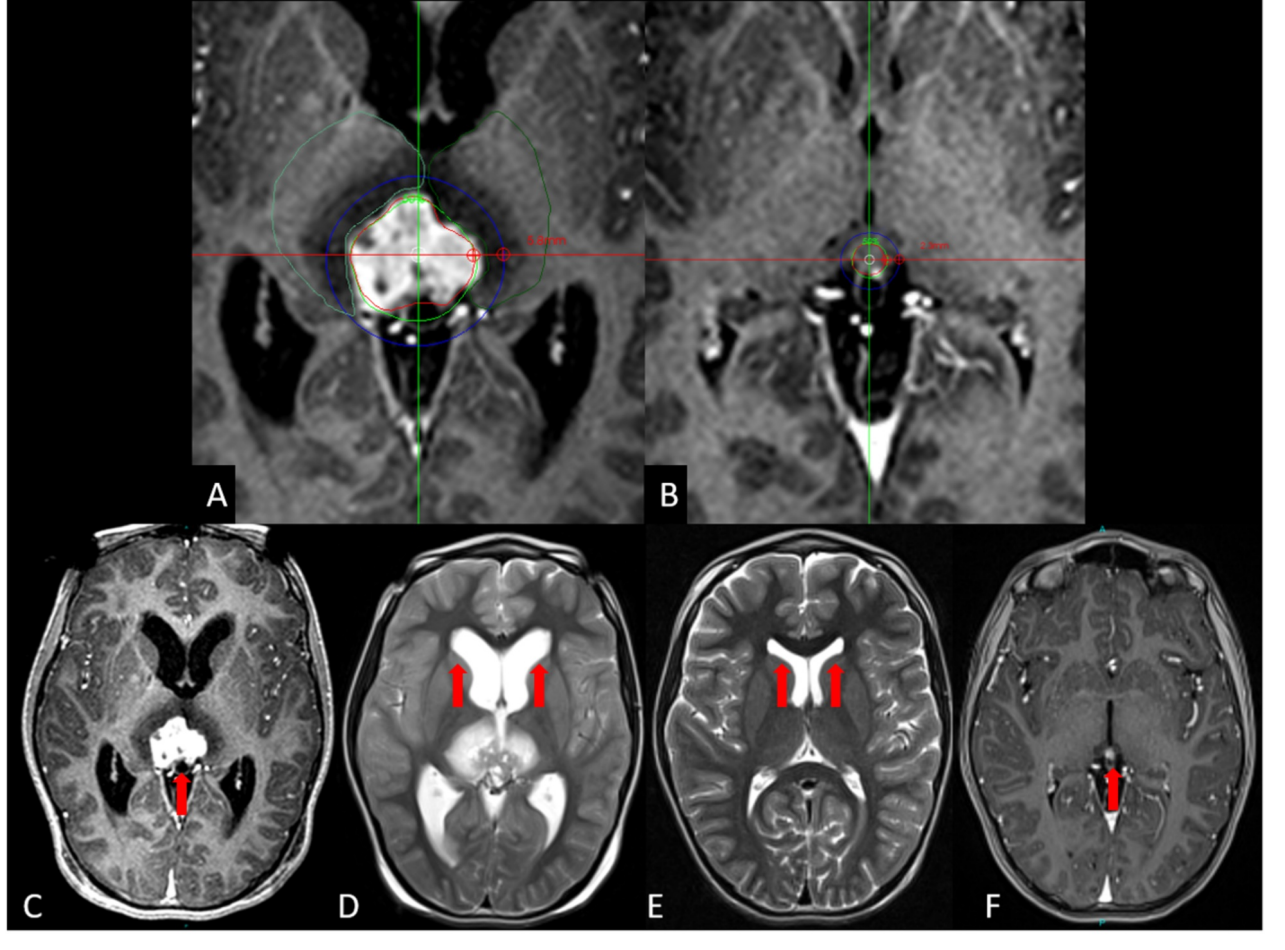
Patient six presenting with a pinealocytoma A. Close up, axial T1 gadolinium-enhanced image of the SRS plan. The tumor volume is being covered by the 50% isodose line in green during the first session. The prescription dose was 13 Gy. The blue line corresponds to a dose gradient of 6.5 Gy measuring 5.8 mm in healthy tissue. B. A second session 42 days later with an important reduction (98.6%) of the lesion volume. A new target is being prescribed 10 Gy in the second session, to the 50% isodose line in green. The blue isodose line corresponds to the new dose gradient of 2.3 mm to half of the prescription dose. C. Red arrow signals the original lesion. D and E the T2 axial images between the first and the second treatment respectively, in both images the red arrows point to the anterior ventricle horns showing acute hydrocephalous in D and in E a complete resolution of the hydrocephalous without shunt placement. F. T1 gadolinium-enhanced image at six months showing the tumor that is signaled by the red arrow that remains stable. SRS, stereotactic radiosurgery.

**Figure 3:**
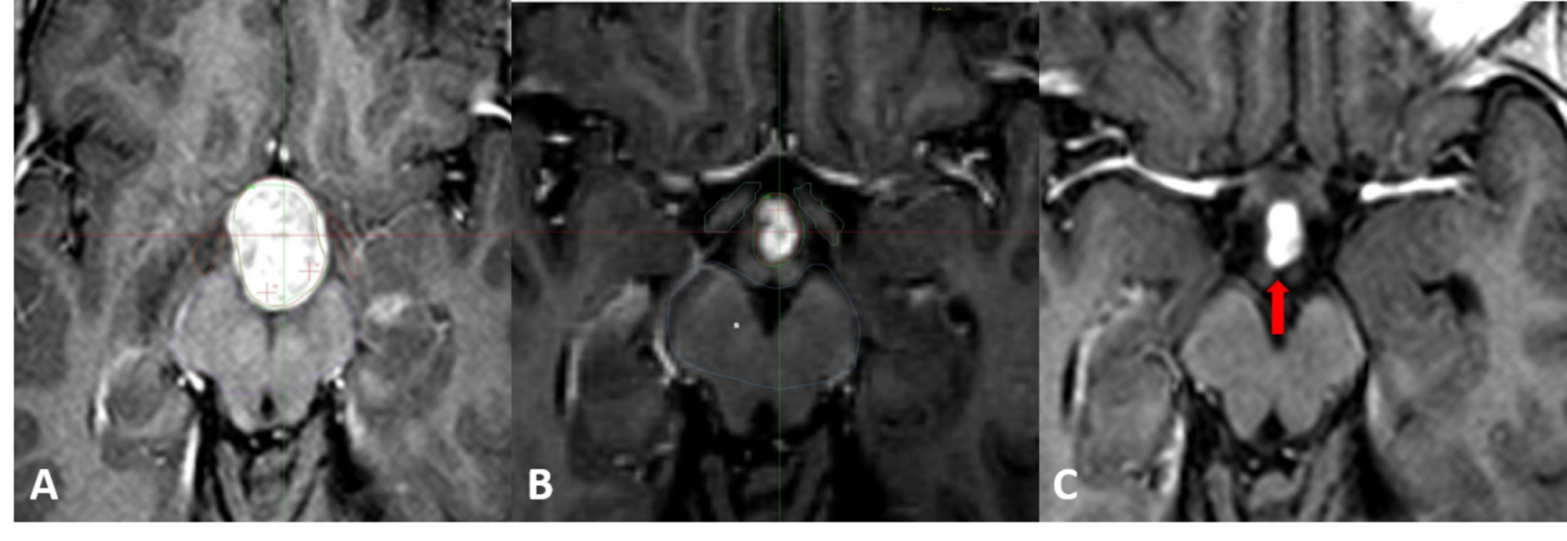
Patient one presenting a drop metastasis from a posterior fossa medulloblastoma A. The radiosurgical plan with T1 gadolinium-enhanced imaging of the tumor located in the hypothalamic region. The initial tumor volume was 5.2 ml and was prescribed 10 Gy to the 50% isodose line. B. At 30 days, during the second radiosurgical plan, the T1 gadolinium-enhanced image shows a reduction of 92.3% of the tumor volume; it now measures 0.4 ml and was prescribed 13 Gy to the 50% isodose line. C. T1 gadolinium-enhanced imaging taken at three months after the second session of radiosurgery, the lesion remains stable as signaled by the red arrow.

At last follow-up, at a mean time of 336.5 days (range, 45 to 962), seven patients were alive. Five tumors had a partial response (PR), while three had stable disease (SD) in accordance with the RECIST scale (Figures 2 & 3).

The one patient that died (patient one) passed away 435 days after treatment due to leptomeningeal disease. Patient five with the germinoma tumor developed peritoneal dissemination of the disease due to a ventricular peritoneal shunt but was alive at last follow-up. The Karnofsky Performance status was 90 (range 20 to 90) at the first session, and 90 (range 50 to 100) at the second session; the status was maintained at last follow-up. In patient two (Figure 2) and patient seven, symptomatic secondary hydrocephalous by the tumor mass was present. Shunt placement could be avoided by quick tumor response to radiosurgery. No adverse radiation effects were reported.

## Discussion

Current dose recommendations for single-session radiosurgery by multiple series for primary brain tumors hovers around 15 Gy for most series [17-22]. As the allowable value depends on tumor volume or the organs at risk abutting the lesion, this is not always possible. Lowering the prescription dose to comply with the organs at risk tolerance could potentially sacrifice long-term tumor control. Hypofractionation is a possible alternative for dose escalation.

Nevertheless, our group (unpublished data) has developed comparative studies between hypofractionation versus two-session radiosurgery in brain metastases. These data indicate an expected similar tumor alpha-beta that demonstrates potential advantages in favor of two-session therapy for healthy tissue sparring at equivalent BED with regard to hypofractionation. The main reason is attributed to tumor shrinkage during the 30-day time interval during each session; a smaller lesion allows a sharper dose gradient than larger ones; this is illustrated in Figure 1, and Figure 2 shows how the dose gradient and volume changes occur over 30 days. In our series of metastatic tumors from the breast and lung, mean tumor volume reduction between each session has been 66% and 73% in the current series; only one lesion in the anaplastic ependymoma case showed tumor enlargement; it did not exceed 20% [1].

In our comparative studies when hypofractionation was done for three fractions, to reach the same equivalent dose of two-session therapy, the volume of healthy tissue (dose gradient) is unaltered during treatment. The PTV remains a consistent volume as the same plan is executed without modifications during hypofractionation; so, depending on the initial size of the lesion, this traduces into potentially three to four-fold more healthy tissues receiving high doses of radiation than a two-session SRS. Further comparative studies regarding adverse effects from radiation and local tumor control are necessary between both radiosurgical techniques.

The hypothalamus is particularly challenging for any radiosurgery modality due to the crucial structures involved in and surrounding this area, such as the peduncles and, more importantly, the intracranial portion of the optic pathway. In these tumors and in large tumors in the pineal region that displace the thalamus and the mesencephalic tectum, we draw a PTV inside the GTV as specified and shown in Figure 1. This explains the relatively low coverage of the PTV. We used a similar technical concept as surgical debulking, consisting of intracapsular tumor removal to carefully separate the lesion from vital structures and allow safe removal. The same philosophy is intended when prescribing a higher dose to a smaller PTV than the actual GTV as it is expected to retract the tumor away from the organs at risk during the process of tumor shrinkage, to allow a higher dose during the second session of radiosurgery.

Dose heterogeneity and a higher mean dose when the treatment prescription is delivered to the 50% isodose line might be relevant in tumor control as noted by Shaw while prescribing the same dose favoring Gamma Knife over a linear accelerator with regards to tumor control [9]. It is possible that the higher intratumor mean dose in the types of tumors treated in this series favors prompt symptom relief, including those caused by hydrocephalous such as patient two and seven where a ventriculoperitoneal shunt surgery was avoided. Further studies are necessary to validate if a higher mean dose is relevant for quicker relief of neurological symptoms by tumor mass effect. It also needs to be determined if a higher mean dose is relevant for longer tumor control in primary brain tumors, as it was demonstrated in brain metastases.

Two- and three-stage SRS has been proven to be a safe dose escalation protocol in large brain metastases. It may be helpful in primary brain tumors that, by histology, are expected to respond in a similar fashion, as demonstrated in this initial series. Nevertheless, there is very limited experience at this time, and larger multicentric studies are necessary to understand the role of this modality in the modern management of primary brain tumors.

## Conclusions

Two-stage SRS proved to be a safe method to escalate dose in proportionately large volume primary brain tumors whose histology is expected to have a quick biological response to radiation. In this small series and in very select cases two-session SRS proved to be an option to resolve hydrocephalous without the need of ventriculoperitoneal shunt placement. Longer follow-up is needed to determine the long term effectiveness by tumor subtypes of two-stage SRS in the same manner as it has been proven in single session SRS series in smaller tumor volumes.
